# Comparing the Efficacy Between Entry-Level Torsional and High-End Transverse Phacoemulsification Technologies

**DOI:** 10.7759/cureus.106918

**Published:** 2026-04-12

**Authors:** Naren Shetty, S J Tejal, Rohitha Nayak, Akash Jain, Nisarga Rane, Chaitra Jayadev

**Affiliations:** 1 Cataract and Refractive Surgery, Narayana Nethralaya, Bangalore, IND; 2 Cataract, Narayana Nethralaya, Bangalore, IND; 3 Vitreoretina Services, Narayana Nethralaya, Bangalore, IND

**Keywords:** cataract, efficacy, entry-level torsional system, phacoemulsification, transverse phacoemulsification technology

## Abstract

Clinical relevance: With the growing cost of equipment, uncompromised clinical care is paramount. The best possible scenario is an affordable device that delivers optimal results. Clinical studies demonstrating comparability between low-cost and high-end devices can help clinicians make informed decisions.

Background: To compare the intra-operative efficacy of two phacoemulsification technologies - torsional and transverse.

Methods: This was a prospective comparative study with systematic (quasi-random) allocation conducted at a tertiary eye care center. The study included 120 eyes from 112 patients with immature cataracts. Phacoemulsification was performed using either an entry-level torsional (Legion; Alcon Laboratories, Inc., Fort Worth, TX) or a high-end transverse (WhiteStar Signature Pro; Johnson & Johnson Vision, Inc., New Brunswick, NJ) machine by the same surgeon in patients assigned into two groups of 60 eyes each. Routine biometry, preoperative and postoperative day (POD 1, 30, and 90) examination on specular microscopy, pachymetry, and best-corrected visual acuity assessment were done. Intraoperatively, the mean surgical time and ultrasound time were noted. An independent, masked evaluator assessed the surgical videos.

Results: The torsional system required 47.12% less ultrasound time (p < 0.001) and 5.15% less surgical time (p = 0.056) than the transverse system, with less chatter, better followability, and easier quadrant removal. There was no significant difference in postoperative visual outcomes or pachymetry measurements between preoperative and POD 1 or POD 30 in either group. Similarly, endothelial cell density changes from pre-op to POD 1, 30, and 90 were not statistically significant in both groups.

Conclusion: The torsional phacoemulsification system required less ultrasound energy and a marginally shorter mean surgical time than the transverse system. The ease and efficiency of phacoemulsification, as measured by reduced chatter and better quadrant removal, were also better with the more affordable entry-level torsional system.

## Introduction

Phacoemulsification has revolutionized cataract management by enhancing surgical efficiency, precision, and predictability, thereby resulting in superior postoperative visual outcomes [1]. Despite these advances, optimizing ultrasound energy delivery and intraoperative safety remains a major focus of technological innovation. With the increasing global burden of visually significant cataracts, particularly in resource-constrained settings, there is a growing need for cost-effective phacoemulsification platforms that can deliver optimal outcomes while minimizing ultrasound energy, surgical time, and associated complications, such as cystoid macular edema, wound burns, and corneal endothelial cell loss [2,3].

Recent developments in phacoemulsification technology have emphasized reducing ultrasound energy dissipation to achieve more controlled nuclear emulsification while preserving intraocular structures. Conventional phacoemulsification systems employ longitudinal ultrasound, in which the phaco tip moves in a forward-and-backward direction at a fixed frequency. This mechanism often leads to repulsion of nuclear fragments, termed "chatter," which reduces followability and efficiency of emulsification [4]. Furthermore, cavitation bubble formation, previously considered advantageous, has now been implicated in generating free radicals, increasing intraocular turbulence, and potentially causing collateral tissue damage [5].

To address these limitations, alternative ultrasound modalities have been introduced. Torsional ultrasound technology employs a rotary oscillatory movement of the phaco tip, facilitating nuclear disassembly through a shearing mechanism rather than forward propulsion, thereby reducing chatter and improving followability [6]. In contrast, transverse ultrasound systems utilize a lateral side-to-side motion of the phaco tip, which also enhances cutting efficiency while reducing thermal energy generation and intraocular turbulence [7]. Both modalities have been successfully integrated into contemporary phacoemulsification platforms and have demonstrated improved intraoperative performance compared to conventional longitudinal systems.

In addition to technological differences, economic considerations play a critical role in the adoption of phacoemulsification systems, particularly in developing countries such as India. The Alcon Legion system, an entry-level platform equipped with torsional ultrasound technology, is relatively more affordable, with an estimated cost of approximately two million Indian rupees. In contrast, the WhiteStar Signature Pro system, a higher-end platform incorporating transverse ultrasound technology along with advanced fluidics and software integration, is typically priced higher, at approximately five million Indian rupees. This cost differential may influence purchasing decisions in high-volume surgical centers and necessitate evaluating whether higher capital investment translates into clinically meaningful improvements in surgical efficiency and patient outcomes.

Another important factor influencing surgical outcomes is the operating surgeon's experience and skill. Surgeon-related variables, including familiarity with machine-specific fluidics, ultrasound modulation, and phaco techniques, can significantly impact intraoperative efficiency, ultrasound time, and complication rates. While advanced technologies may offer theoretical advantages, their real-world performance is often modulated by the surgeon's learning curve and adaptability to different platforms.

In this context, we hypothesize that, although both phacoemulsification systems are safe and effective, there may be significant differences in intraoperative efficiency - particularly in terms of mean surgical time and ultrasound energy utilization - as well as postoperative outcomes. Furthermore, we aim to assess whether these differences justify the cost disparity between the two platforms. Accordingly, this study was designed to compare the entry-level Legion system, utilizing torsional ultrasound technology, with the high-end WhiteStar Signature Pro system, employing transverse ultrasound technology, with respect to intraoperative efficiency and postoperative outcomes.

## Materials and methods

The study was a prospective single-center comparative study with systematic (quasi-random) allocation, approved by the Institutional Ethics Committee and performed in accordance with the tenets of the Declaration of Helsinki. After informed consent, 120 eyes of 112 patients were included, 60 in each group. The primary outcome measure was intraoperative ultrasound time. Secondary outcomes included total surgical time and endothelial cell loss. Hence, for the primary outcome measure of intraoperative ultrasound time, a sample size estimate was performed for this two-group study with an acceptable alpha error of 5% and 80% power, yielding a calculated sample size of 50 per group. Considering a 10% dropout rate, the final sample size was 60 per group.

All underwent a complete ophthalmic examination, including visual acuity assessment, manifest refraction, slit lamp evaluation, and fundoscopy. Patients aged 40-75 years with cataracts of grade NS2 and NS3 according to the Lens Opacities Classification System III (LOCS III) classification with normal biometry (keratometry range: 41-46 D, axial length range: 21-26 mm, intraocular power range: +19 to +26 D) and intraocular pressure within the normal range were recruited. The exclusion criteria were as follows: subjects with complicated cataracts secondary to trauma/subluxated cataract, posterior polar cataract, floppy iris syndrome, status post any retina surgery, or anti-vascular endothelial growth factor (VEGF) injection, post-uveitis cataract, presence of axial length more than 26 mm or less than 21 mm, presence of corneal dystrophies/corneal scars/opacities, presence of anterior chamber abnormalities like iris coloboma/lens coloboma, peripheral anterior synechiae or peripheral iridotomy, post corneal surgeries such as penetrating keratoplasty or refractive procedures, small pupil, pseudoexfoliation and all types of glaucoma. Patients with an electronic medical record (EMR) number ending in an even number were recruited to the Legion group (Alcon Laboratories, Inc., Fort Worth, TX), and those with an odd number were recruited to the WhiteStar Signature Pro group (Johnson & Johnson Vision, Inc., New Brunswick, NJ) until both groups had a similar number of patients.

A single experienced surgeon performed standard phacoemulsification with a 2.8-mm incision for all patients. The surgery in the Legion group was performed with a constant aspiration flow rate (AFR) of 60, a constant vacuum of 650 mmHg, and a bottle height of 95 cmH2O. In the WhiteStar Signature Pro group, fixed machine parameters, such as a signature linear AFR of 10-60, a linear vacuum of 180-650 mmHg, a linear power of 0-100, and a bottle height of 95 cmH₂O, were used for phacoemulsification. Intra-operative ultrasound time and total surgical time were noted. Postoperatively, subjects were evaluated on days 1, 30, and 90. On day one after surgery, the measurements included uncorrected visual acuity, intraocular pressure, specular microscopy, and corneal pachymetry using an anterior segment optical coherence tomography. A comprehensive slit-lamp exam was performed, including Seidel's test to rule out any wound leak. Corneal pachymetry was done at the one-month visit, along with the routine assessment. The assessment at the three-month follow-up included specular microscopy and routine tests. An independent evaluator assessed the masked surgical video. The surgeries were scored (Appendix 1) for chamber stability (Grades 1-4), surge control (Grades 1-3), chatter (Grades 1-3), followability (Grades 1-3), ease of primary chop/sculpt (Grades 1-4), ease of quadrant removal (Grades 1-3), ease of epinucleus removal (Grades 1-3), and ease of cortex aspiration (Grades 1-3).

Statistical Analysis

Continuous variables were assessed for distribution and summarized as mean ± standard deviation (SD) or median (interquartile range, IQR) as appropriate. Between-group comparisons were performed using the independent samples t-test for approximately normally distributed variables and the Mann-Whitney U test for non-normal distributions. Categorical variables were compared using the chi-square test or Fisher's exact test. For corneal pachymetry and endothelial cell density, both absolute change (POD-pre) and relative change (% from baseline) were calculated. A multivariable linear regression model was additionally constructed with endothelial cell loss at POD 90 as the dependent variable and machine type, ultrasound time, cataract grade (NS2/NS3), and age as covariates. A two-sided p-value <0.05 was considered statistically significant.

## Results

Both groups had similar patient ages and baseline characteristics. Among the 91 NS2 cataract cases, 45 were treated with the torsional or Legion and 46 with the transverse or WhiteStar Signature Pro phacoemulsification system. Of the 29 eyes graded as NS3, 15 were operated on using the torsional phacoemulsification system and 14 using the transverse phacoemulsification system. Follow-ups for all patients were completed as per protocol. In terms of surgical efficiency, the median value (95% Cl) of mean intraoperative ultrasound time (UST) was 25.83 (19.8-31.8) seconds in the Legion group, compared to 48.84 (41.9-55.6) seconds with the WhiteStar Signature Pro system, with the Legion taking 47.12% less UST (p < 0.001, Table 1). The mean surgical time was 322.73 (305.631-339.836) seconds in the Legion group and 340.25 (320.869-359.631) in the WhiteStar Signature Pro group (Table 1). A subgroup analysis by nuclear grade with NS2 (Legion n = 45 vs Signature n = 46) and NS3 (Legion n = 15 vs Signature n = 14) showed a significant difference only in mean UST: 23.16 vs 43.63 (p < 0.001) and 33.85 vs 65.96 (p < 0.001), respectively. No intraoperative complications were noted with both machines.

**Table 1:** Comparing the median ultrasound time and mean case time in seconds between the two machines

Median (95% CI)	Legion	Whitestar Signature Pro	p-value
Median ultrasound time (seconds)	25.83 (19.8, 31.8)	48.84 (41.9, 55.6)	<0.001
Median surgical time (seconds)	322.73 (305.631, 339.836)	340.25 (320.869, 359.631)	0.06

The difference in pachymetry between POD 1 and pre-operative value in the Legion group was 13.0 (11.0-16.0) μm, and in the WhiteStar Signature Pro group, 14.0 (9.0-21.0) μm (p = 0.1, Table 2). The drop in endothelial cell density on POD 90, when compared to pre-operative values, was 209.5 (114.817-277.548) in the Legion group and 183 (164.270-278.548) in the WhiteStar Signature Pro group (p = 0.5, Table 2). The relative change for CCT for POD1-pre (μm) was median 12 vs 15 (p = 0.717), and that for endothelial loss POD90-pre (cells/mm²) was median 241.5 vs 176.5 (p = 0.354).

**Table 2:** Comparing the median increase in central corneal thickness postoperatively and specular microscopy between the two machines

Median (95% CI)	Legion	Whitestar Signature Pro	p-value
Difference in central corneal thickness between postoperative day 1 and preoperative	13.0 (11.0-16.0)	14.0 (9.0-21.0)	0.1
Difference in specular microscopy between preoperative and postoperative day 90	209.5 (114.817-277.548)	183 (164.270-278.548)	0.5

A comparison of endothelial cell density on POD 30 with preoperative values showed a greater reduction in the Legion group than in the Signature Pro group. A similar trend was observed on POD 1, with a higher cell loss in the Legion group. However, the difference in cell loss between the two groups was not statistically significant. In multivariable regression, adjusting for UST, cataract grade, and age, machine type was not an independent predictor of endothelial cell loss at POD 90, whereas higher UST was associated with greater endothelial loss.

Based on an independent evaluator's masked assessment of surgical videos, the Legion group was found to have less chatter (p < 0.001) and greater ease of quadrant removal (p = 0.006) than the WhiteStar Signature Pro group. There was no significant difference between the two groups for ease of followability, chamber stability, surge control, ease of primary chop/sculpt, ease of epinucleus removal, and ease of cortex aspiration.

## Discussion

Although considered an entry-level device, the Legion system incorporates torsional Ozil ultrasound technology, designed to reduce heat generation and improve followability by reducing the repulsion of nuclear fragments. The 45-degree-bent Kelman balanced tip may further contribute to cutting precision and facilitate smoother emulsification with potentially lower energy dispersion. In comparison, the WhiteStar Signature Pro employs Ellips FX technology, combining transverse and longitudinal ultrasound to enhance cutting efficiency; however, this approach may be associated with increased fragment repulsion in certain settings. The WhiteStar Signature Pro also offers a dual-pump system (peristaltic and venturi), providing greater flexibility in fluidics management. Overall, both systems demonstrate distinct technological approaches to phacoemulsification. The Legion system may offer advantages in terms of torsional efficiency and reduced repulsion, whereas the WhiteStar Signature Pro provides versatility in fluidics control. The choice between the two platforms may therefore depend on surgical preference, case requirements, and cost considerations.

In our study, the Legion group had significantly lower intraoperative ultrasound time and total surgical time. The literature highlights that pivotal factors influencing favorable postoperative visual outcomes and corneal clarity are the total duration and magnitude of phacoemulsification energy applied during the surgical procedure [8]. A comprehensive analysis of surgical videos underscores the Legion's superiority, with less chatter, improved followability, and easier quadrant removal, even at high-power settings, due to its minimal repulsive effect compared to transverse technology [9].

Minimizing endothelial damage is crucial in phacoemulsification, as excessive intraocular manipulation and ultrasonic energy from the phacoemulsification tip can potentially lead to iatrogenic cell loss [10]. Although endothelial cell loss was higher in the Legion group, the difference between the two groups was not statistically significant. In multivariable regression, adjusting for ultrasound time, cataract grade, and age, machine type was not an independent predictor of endothelial cell loss at POD 90, whereas higher ultrasound time was associated with greater endothelial loss. This strengthens the observation that endothelial safety is influenced more by energy delivery than by the phaco platform itself. A study examining corneal endothelial morphology and function following torsional and longitudinal ultrasound-mode phacoemulsification concluded that both techniques were safe and effective [11]. It was also determined that the torsional handpiece's oscillatory motion delivers less energy to the eye than longitudinal ultrasound, providing a safer energy and thermal profile. Our study corroborated these findings. Another study demonstrated the safety and equivalence of both torsional and transverse phacoemulsification platforms, resulting in minimal corneal changes through a 2.2 mm incision [12].

This study compares an entry-level phacoemulsification machine with a high-end one, and the findings can help surgeons make an informed decision while procuring new equipment. A few limitations of the study include a small sample size, a single surgeon, and a limited range of lens opacities (NS2 and NS3); metrics such as cumulative dissipated energy and phaco energy used were not studied. Studying outcomes in patients with advanced cataracts will provide further insights into the performance differences between these two phacoemulsification machines. Additionally, our study may be underpowered to detect smaller differences in secondary outcomes such as mean case time and endothelial cell loss. There is a potential for allocation bias, but importantly, baseline demographic and clinical characteristics between the two groups were comparable, reducing the likelihood of significant selection bias.

## Conclusions

In conclusion, both the Legion and Whitestar Signature Pro phacoemulsification systems are safe and effective. The Legion phacoemulsification machine had a significantly lower ultrasound time than the Whitestar Signature Pro, with a marginally lower mean surgical time. The ease and efficiency of phacoemulsification, as measured by less chatter, better followability, and quadrant removal, were better with the Legion. This enhanced efficiency translates into reduced surgical time, with minimal corneal changes, making it comparable to high-end phacoemulsification systems. With the rising cost of equipment, maintaining high-quality clinical care is paramount. The ideal scenario involves an affordable device that delivers optimal outcomes. Clinical studies demonstrating comparability between low-cost and high-end devices can help clinicians make informed decisions.

## Appendices

Appendix 1: Grading Used for Various Steps During Surgery Using the Two Phacoemulsification Machines

Chamber stability:

Grade 1: Shallowing of the anterior chamber noted in ≥ 3 steps of phacoemulsification

Grade 2: Shallowing noticed in any two steps during surgery

Grade 3: Shallowing noticed during any one step during surgery

Grade 4: Chamber stable throughout with no shallowing at any given point during the surgery

Surge control:

Grade 1: Exaggerated shallowing of the anterior chamber during nucleus impalement

Grade 2: Shallowing noted during impalement, followed by quick recovery with formation of the anterior chamber

Grade 3: Well-formed anterior chamber during impalement and emulsification of the nucleus

Chatter:

Grade 1: Noticeable nuclear fragments repelling against the phacoemulsification tip during the entire period of nuclear quadrant removal

Grade 2: Noticeable nuclear fragments repelling while emulsification of one nuclear segment

Grade 3: No chatter observed

Followability of nuclear pieces:

Grade 1: Noticeable excursion of the phacoemulsification tip away from the safe central zone during nuclear pieces removal and difficulty in cortex aspiration

Grade 2: Difficulty noticed during either one of the steps, nucleus removal or cortex aspiration

Grade 3: Good followability of both nuclear and cortical material

Ease of primary chop/sculpt:

Grade 1: Unsuccessful impalement for effective chopping

Grade 2: Impalement achieved, but difficulty in chopping

Grade 3: Impalement successful, the nucleus splits, but the depth of chop is variable

Grade 4: Smooth impalement and complete nuclear separation with a single chop

Ease of quadrant removal:

Grade 1: Difficulty in impaling and manipulating nuclear quadrants

Grade 2: Difficulty in removing 1-2 nuclear quadrants

Grade 3: Smooth removal of all the quadrants

Ease of epinucleus removal:

Grade 1: Inability to remove the epinucleus despite multiple attempts

Grade 2: Epinucleus removed with difficulty or as piecemeal

Grade 3: Epinucleus removal was smooth and was removed in toto

Ease of cortex aspiration:

Grade 1: Unstable anterior chamber with difficulty in engaging and aspiration of cortex

Grade 2: Cortex removal without any difficulty in all the quadrants except in the subincisional area

Grade 3: Cortex aspiration occurs smoothly
